# Dopamine D1 Receptor Immunoreactivity on Fine Processes of GFAP-Positive Astrocytes in the Substantia Nigra Pars Reticulata of Adult Mouse

**DOI:** 10.3389/fnana.2017.00003

**Published:** 2017-02-01

**Authors:** Katsuhiro Nagatomo, Sechiko Suga, Masato Saitoh, Masahito Kogawa, Kazuto Kobayashi, Yoshio Yamamoto, Katsuya Yamada

**Affiliations:** ^1^Department of Physiology, Hirosaki University Graduate School of MedicineAomori, Japan; ^2^Department of Emergency Medical Technology, Hirosaki University of Health and WelfareAomori, Japan; ^3^Laboratory of Veterinary Anatomy and Cell Biology, Faculty of Agriculture, Iwate UniversityIwate, Japan; ^4^Department of Molecular Genetics, Institute of Biomedical Sciences, Fukushima Medical University School of MedicineFukushima, Japan

**Keywords:** glia, dendritic release, basal ganglia, striatum, visual cortex

## Abstract

Substantia nigra pars reticulata (SNr), the major output nucleus of the basal ganglia, receives dopamine from dendrites extending from dopaminergic neurons of the adjacent nucleus pars compacta (SNc), which is known for its selective degeneration in Parkinson's disease. As a recipient for dendritically released dopamine, the dopamine D1 receptor (D1R) is a primary candidate due to its very dense immunoreactivity in the SNr. However, the precise location of D1R remains unclear at the cellular level in the SNr except for that reported on axons/axon terminals of presumably striatal GABAergic neurons. To address this, we used D1R promotor-controlled, mVenus-expressing transgenic mice. When cells were acutely dissociated from SNr of mouse brain, prominent mVenus fluorescence was detected in fine processes of glia-like cells, but no such fluorescence was detected from neurons in the same preparation, except for the synaptic bouton-like structure on the neurons. Double immunolabeling of SNr cells dissociated from adult wild-type mice brain further revealed marked D1R immunoreactivity in the processes of glial fibrillary acidic protein (GFAP)-positive astrocytes. Such D1R imunoreactivity was significantly stronger in the SNr astrocytes than that in those of the visual cortex in the same preparation. Interestingly, GFAP-positive astrocytes dissociated from the striatum demonstrated D1R immunoreactivity, either remarkable or minimal, similarly to that shown in neurons in this nucleus. In contrast, in the SNr and visual cortex, only weak D1R immunoreactivity was detected in the neurons tested. These results suggest that the SNr astrocyte may be a candidate recipient for dendritically released dopamine. Further study is required to fully elucidate the physiological roles of divergent dopamine receptor immunoreactivity profiles in GFAP-positive astrocytes.

## Introduction

The midbrain nucleus substantia nigra pars reticulata (SNr) consists mostly of gamma-aminobutyric acid-ergic (GABAergic) neurons. GABAergic SNr neurons receive inhibitory and excitatory axonal inputs from striatum and subthalamus, respectively, and send in turn their axons to remote nuclei, such as superior colliculus, thalamus, and brain stem (Fallon and Loughlin, 1995).

It is well accepted that dopaminergic neurons located in the adjacent nucleus substantia nigra pars compacta (SNc) release dopamine (DA) from their dendrites (dendritic release) extending deeply into SNr (Geffen et al., 1976; Korf et al., 1976; Nieoullon et al., 1977; Reubi et al., 1977; Cheramy et al., 1981; Falkenburger et al., 2001). Whereas, the significance of dopamine released from nigrostriatal axons has been extensively studied both physiologically and pathophysiologically, much less attention has been paid to dopamine dendritically released in the SNr, in particular to the cellular entity expressing dopamine receptors (Dunnett and Bjorklund, 1999; Schultz, 2015; Volkow and Morales, 2015).

Of five dopamine receptor subtypes, it has been reported that dopamine D1 receptor (D1R) is strongly expressed in the SNr, whereas expression of dopamine D2 receptor (D2R) is relatively sparse in this nucleus (Levey et al., 1993; Fallon and Loughlin, 1995).

Regarding the precise localization of D1R in the SNr, several groups have provided evidence for its expression on axons and axon terminals of GABAergic neurons that may have originated in the striatum (Levey et al., 1993; Fallon and Loughlin, 1995; Yung et al., 1995; Caille et al., 1996; Miyazaki and Lacey, 1998; Kliem et al., 2010). However, the very dense D1R immunoreactivity in SNr (Levey et al., 1993; Caille et al., 1996) led us to explore further whether other cellular entities might also contribute to the expression.

Over 30 years ago, Reubi and Sandri reported in electronmicroscopic/freeze etching studies that nigral dendrites fail to form dendro-dendritic contacts in the SNr, but are consistently separated by one or two thin glial sheaths (Reubi and Sandri, 1979). Interestingly, Bosson et al. reported that acute interruption of dopaminergic transmission increased astrocyte synchrony in the SNr (Bosson et al., 2015).

Thus, as the major glial cell, the astrocyte might well express D1R on its thin processes in the SNr. To address this, we initially conducted double immunolabeling of SNr slices with antibodies for D1R and 3-PGDH (3-phosphoglycerate dehydrogenase; Yamasaki et al., 2001), a marker that can stain fine processes of astrocytes that was used here instead of an antibody against intermediate filament glial fibrillary acidic protein (GFAP). However, identification of the D1R-positive cellular entity using confocal microscopy was difficult due to its extremely fine and dense pattern in the SNr, although considerable overlapping of the immunoreactivity for D1R and 3-PGDH was apparent.

We therefore examined acutely dissociated cells from D1R promoter-controlled, mVenus (a variant of enhanced yellow fluorescent protein)-expressing transgenic (Drd1-mVenus) mice brain (Nagai et al., 2016). For further identification of mVenus-positive cells, double immunolabeling with antibodies against D1R and GFAP was conducted in the SNr of adult wild-type mice brains. For comparison, cells dissociated from the visual cortex and the striatum also were analyzed.

Our results suggest involvement of astrocytes in D1R immunoreactivity in the SNr and heterogeneity of astrocytes in adult mouse brain.

## Materials and methods

### Animals

Animals used in the present study were male C57BL/6J mice, mice lacking D1R (D1R-KO mice; RBRC01080; Tran et al., 2008; Nakamura et al., 2014), and Drd1-mVenus mice (RBRC03111). Drd1-mVenus mice were developed by one of the authors of the present study (KK, see Nagai et al., 2016 for detail). The D1R-KO mice and Drd1-mVenus mice used were obtained from RIKEN BioResource Center (Tsukuba, Japan). Their genotypes were determined by PCR analysis of genomic DNA extracted from the tail of each mouse.

Both the D1R-KO mice and Drd1-mVenus mice were generated on a C57BL/6J background. D1R-KO mice were obtained by crossing heterozygous D1R-KO mice. Homozygous Drd1-mVenus transgenic mice were obtained by crossing heterozygous Drd1-mVenus transgenic mice. Determination of homozygosity of Drd1-mVenus mice was made based on the genotype of 8 consecutive pups produced by crossing presumptive homozygous male (or female) mice with female (or male) C57BL/6J mice. These transgenic mice expressed mVenus in a D1R-promotor-dependent manner.

All animal experiments were carried out in accordance with a protocol approved by the Animal Care and Use Committee of Hirosaki University Graduate School of Medicine.

### Brain slice preparation for immunohistochemistry

Four adult D1R-KO and ten wild-type mice (39–47 days old) were deeply anesthetized with urethane (1.6 g/kg) and perfused through the heart with 4% paraformaldehyde in 0.1 M phosphate buffer (pH 7.4) following 0.1 M phosphate buffer (pH 7.4) containing heparin (10,000 units/l, Mochida Pharmaceutical, Tokyo, Japan) at room temperature. After post-fixation at 4°C, the brains were dissected out, and coronal or sagittal sections were then cut with cryostat at 50 μm in thickness and were processed for immunohistochemistry using the avidin-biotin-peroxidase complex method. Briefly, the sections were treated with Phosphate buffered saline (PBS) containing 0.5% Triton X-100 for 12 h at 4°C, with 0.3% H_2_O_2_ in PBS for 1 h at room temperature, and with 1% normal donkey serum in PBS for 1 h at room temperature. Washing previous solutions with PBS was done between each treatment. The sections then were treated with a primary antibody for 48 h at 4°C, with a biotinylated secondary antibody for 1 h at room temperature and then with an avidin-biotin peroxidase complex (2.5 μl/ml, Elite ABC kit, Vector, Burlingame, CA, USA) for 1 h at room temperature, with washing between each treatment. After washing, sections were visualized with 0.05 M Tris-HCl buffer (pH 7.4) containing 0.02% 3-3′-diaminobenzidine tetrahydrochloride (DAB) and 0.006% H_2_O_2_ for 15–30 min at room temperature. The sections were mounted on glass slides, embedded, and examined with a light microscope (BX-50, Olympus, Tokyo, Japan).

### Preparation from Drd1-mVenus mice

Five Drd1-mVenus male mice (47–72 days old) were deeply anesthetized by urethane (1.6 g/kg) and sacrificed by decapitation. The brain was quickly isolated from the skull, and a 500 μm-thick coronal slice containing both SNr (a section at the oculomotor nerve) and visual cortex was then dissected out. Slices were recovered for 1 h in a Krebs-Ringer solution containing 124 mM NaCl, 26 mM NaHCO_3_, 4 mM KCl, 1.24 mM KH_2_PO_4_, 1.3 mM MgSO_4_·7H_2_O, 1 mM CaCl_2_, 10 mM glucose, saturated with 95% O_2_/5% CO_2_ (pH 7.4) at room temperature. Two Drd1-mVenus mice were used for examining the mVenus signal throughout the brain in coronal sections. Fluorescence images were captured by a CCD camera (Retiga 2000R, QImaging, Surrey, British Columbia, Canada) and mounted on an inverted microscope (Eclipse Ti-E, Nikon, Tokyo) with a standard FITC filter set.

The remaining Drd1-mVenus mice were used for cell dissociation. In HEPES buffer solution (150 mM NaCl, 5 mM KCl, 1 mM MgCl_2_, 2 mM CaCl_2_, 10 mM HEPES, 10 mM glucose; pH was adjusted at 7.33–7.35 by tris hydroxymethyl aminomethan), single neurons and glia-like cells were dissociated from a small piece of SNr tissue by slightly modifying our previous method (Yamada et al., 2001) so that the thin processes of the glia-like cells were preserved as far as possible. SNr and SNc were identified in reference to Paxinos, G. and Franklin, K.B.J. “The mouse brain in stereotaxic coordinates” (Paxinos and Franklin, 2001).

**Figure 1:**
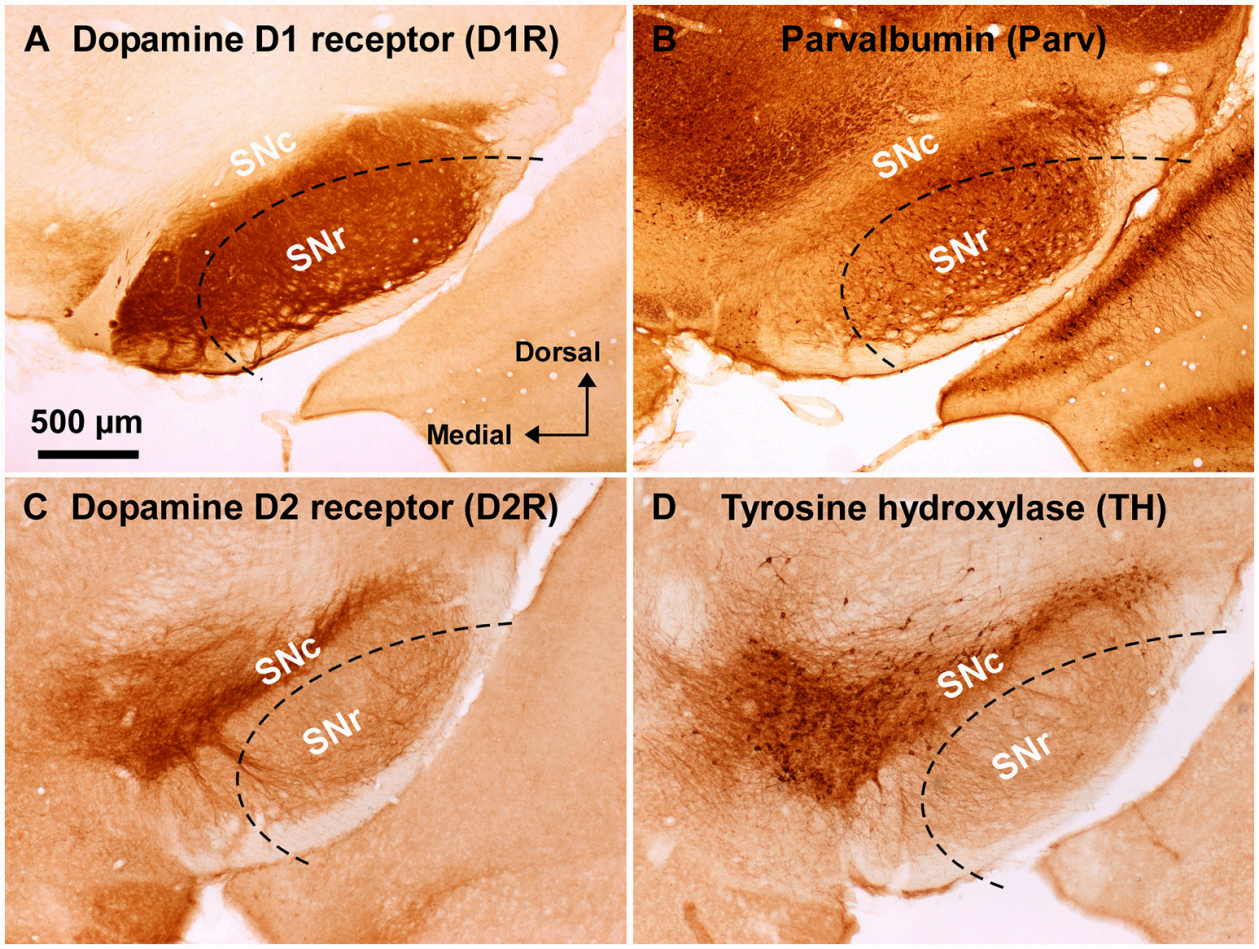
**Immunoreactivity of anti-dopamine D1 and anti-dopamine D2 receptor antibodies used in wild-type mouse substantia nigra**. **(A)** Dense dopamine D1 receptor (D1R) immunoreactivity was detected in substantia nigra pars reticulata (SNr), but not in pars compacta (SNc). **(B)** Abundant GABAergic neurons were shown in the SNr by parvalbumin (Parv) immunoreactivity. **(C)** Dopamine D2 receptor (D2R) immunoreactivity was remarkable in the SNc, but only sparse in the SNr. **(D)** Tyrosine hydroxylase (TH) immunoreactivity was well correlated with D2R immunoreactivity in the substantia nigra. Dashed lines indicate the outer boundary for dissecting out SNr tissue with a customized needle having 200 μm-thick wall. The orientation and scale are common to **A–D**.

Briefly, the dorsolateral region of SNr tissue (Figure 1) was carefully punched out by a customized needle (the major and the minor outer diameter were 1.6 and 1.0 mm, respectively, wall thickness was 200 μm) from a 500 μm-thick coronal slice cut using a microslicer (Linear Slicer Pro7, Dosaka, Tokyo) within the range of 0–0.7 mm anterior to the interaural line, and was subjected to mild digestion by pronase (Calbiochem 537088, 16.7 mg in 100 mL of Krebs-Ringer solution, for 110 min at 31°C). Then cells then were gently dissociated from the tissue and plated on cover glasses (MATSUNAMI; No. 0, 13 × 22 mm) coated with poly-L-lysine (1:500 dilution of 150 mM borate stock solution).

### Antibodies

Primary antibodies used were polyclonal rabbit anti-GFAP antibody (Z0334; 1:2500, DAKO, Carpinteria, CA, USA), monoclonal anti-tyrosine hydroxylase (TH) antibody (MAB318; 1:2000, Millipore, Billerica, MA, USA), rabbit anti-parvalbumin antibody (PV-28; 1:2000, Swant, Marly, Switzerland), goat polyclonal anti-mouse D1R antibody (D1R-Go-Af1000; 1:200, Frontier Institute, Sapporo, Japan; Narushima et al., 2006), and guinea pig anti-mouse D2R antibody (1:1000). The anti-D2R antibody was kindly provided by Prof. Ryuichi Shigemoto (National Institute for Physiological Sciences at that time).

Secondary antibodies used for the avidin-biotin-peroxidase complex method were biotinylated donkey anti-rabbit antibody (711-065-152; 1:1000, Jackson ImmunoResearch Laboratories, West Grove, PA, USA) for parvalbumin, donkey anti-mouse antibody (715-065-151; 1:1000, Jackson ImmunoResearch Laboratories) for TH, donkey anti-goat antibody (705-065-147; 1:1000, Jackson ImmunoResearch Laboratories) for D1R, and biotinylated donkey anti-guinea pig antibody (706-065-148; 1:1000, Jackson ImmunoResearch Laboratories, West Grove, PA, USA) for D2R.

Secondary antibodies used for immunocytochemistry of acutely dissociated cells were Alexa Fluor 488 donkey anti-goat IgG (A-11055; 1:1000, Life Technologies, Waltham, MA, USA) and Cy3-conjugate and donkey anti-rabbit IgG (711-165-152; 1:400, Jackson ImmunoResearch Laboratories, West Grove, PA, USA).

### Immunocytochemistry of cells acutely dissociated from brain of wild-type mice

In a similar manner to the preparation from Drd1-mVenus mice, cells were dissociated from SNr of the brain, but using eight male adult C57BL/6J mice (wild-type, 52–78 days old). For comparison, cells were dissociated from the visual cortex in the same slice using an oval-shaped, customized biopsy needle (the major and the minor outer diameter were 2.9 and 1.2 mm, respectively, wall thickness was 200 μm) so that the gray matter (layer II/III to VI) was included as far as possible.

For a further control, cells were also dissociated from the caudate putamen (striatum). In detail, the brain was first cut coronally into three pieces by dissection about 1.7–1.9 mm anterior to the bregma and about 0–1.1 mm anterior to the interaural line; the middle piece was placed on the stage of the microslicer. A 500 μm-thick coronal slice was dissected out so that it contained the striatum within the range of 1.1–0.3 mm anterior to the bregma. As a result, the slice included the anterior part of the striatum, just rostral to the coronal plane where the anterior commissure crosses the midline. Two or three pieces of punch-out were made by a customized needle (the major and the minor outer diameter were 1.6 and 1.0 mm, respectively) from the striatum in each hemisphere of two male adult wild type mice (46 and 54 days old).

Acutely dissociated cells were fixed with 2% paraformaldehyde in 0.1 M sodium phosphate buffer (pH 7.2–7.4) for 10 min at room temperature, and washed 3 times with PBS for 5 min each. Fixed cells were incubated with PBS containing 0.1% Triton X-100 for 30 min at room temperature, and washed 3 times with PBS for 5 min each. After blocking with PBS containing 10% normal donkey serum (S30-100ML; Millipore, Temecula, CA, USA) for 30 min at room temperature, cells were incubated overnight with a mixture of primary antibodies diluted with PBS containing 10% normal donkey serum at 4°C. After washing the primary antibodies 3 times with PBS for 10 min each, they were incubated for 60 min with a mixture of secondary antibodies at room temperature. After washing, nuclear staining was performed with 4′,6-diamidino-2-phenylindole (DAPI) for 10 min at room temperature; cells were then mounted with PermaFluor (Thermo Scientific; Waltham, MA, USA). Cells similarly processed but with no primary antibodies were used as a control.

### Confocal microscopy

A laser confocal microscope (TCS-SP5, Leica Microsystems, Mannheim, Germany) was used for imaging single cells. mVenus images were obtained with an HCX PL APO CS 40x/1.25-0.75 OIL lens, then quantitatively compared in the same experimental condition with the photomultiplier voltage at 480 V, scan speed at 400 Hz, and pinhole size at 5 airy units. Images were taken two times and averaged. Excitation and emission wavelengths were 514 and 524–790 nm, respectively. For D1R immunocytochemistry, Alexa Fluor 488 fluorescence was captured and quantified similarly, but with the photomultiplier voltage at 580 V. Excitation and emission wavelengths used for detection of the Alexa Fluor 488 fluorescence were 488 and 498–538 nm, respectively. For GFAP immunocytochemistry, Cy3 fluorescence was captured in the condition that GFAP was most clearly detected. Excitation and emission wavelengths for detection of the Cy3 fluorescence were 543 and 565–628 nm, respectively. For detecting DAPI fluorescence, excitation and emission wavelengths at 405 and 415–600 nm were used, respectively. Images were averaged three times.

An HC PL APO 20x/0.70 IMM lens was used for low-magnification image acquisition in one case (Supplementary Figure 4). The photomultiplier voltage for detecting Alexa Fluor 488 (D1R) and Cy3 (GFAP) was 680 and 640 V, respectively, in this case.

For quantitative comparison of the fluorescence, a circular region of interest (ROI, diameter = 5 μm) was placed over the proximal processes of glia-like cells and in the cell body for neurons. The mean fluorescence in the ROI was compared among cells after subtraction of the background intensity using the manufacturer's software (LAS AF Lite, Leica Microsystems) and Photoshop (Adobe Systems).

### Statistics

Values are expressed as mean ± S.D. Statistical significance was analyzed by unpaired *t*-test unless otherwise noted. For D1R immunoreactivity of striatal cells, a cluster analysis was conducted by Ward's method (JMP ver.11.2, SAS Institute, Cary, NC, USA).

## Results

Substantia nigra was identified in coronal slices of wild-type mouse brains (Figure 1). Consistently with well established dopamine D1 and D2 receptor expressions in the midbrain (Levey et al., 1993; Fallon and Loughlin, 1995; Caille et al., 1996), the anti-D1R antibody used in the present study demonstrated very dense immunoreactvity for D1R in SNr (Figure 1A; Supplementary Figure 1B; see also Supplementary Figure 3A for quantification), the region where abundant number of parvalbumin-immunopositive cells were detected compared to that in SNc (Figure 1B; Supplementary Figure 1A; see also Supplementary Figure 2B for statistical comparison; Supplementary Figure 3A), in a way somewhat more robust in the dorsolateral part than in the ventromedial part (Figures 1A,B; Supplementary Figures 2B,3A). The intensity of immunoreactivity for D1R was highly correlated with that for parvalbumin in the dorsolateral part of SNr (Supplementary Figures 3A,C; pairwise Pearson correlation, correlation coefficient = 0.808) as reported previously (Gerfen et al., 1985; Rajakumar et al., 1994).

The specificity of the anti-D1R antibody used was further confirmed in the brain of D1R-KO mice, which showed no appreciable immunoreactivity for the anti-D1R antibody (Supplementary Figure 1C). As reported in previous studies (Fallon and Loughlin, 1995; Yung et al., 1995), our preparations showed relatively sparse D2R immunoreactivity in SNr as compared to that in SNc (Figure 1C; Supplementary Figures 3B,3D for quantification), which contained abundant TH-immunopositive dopaminergic neurons dopaminergic neurons (Figure 1D, Supplementary Figure 2A for statistical comparison, *p* < 0.003, unpaired *t*-test). The intensity of immunoreactivity for D2R was strongly correlated with that for TH in the SN (Supplementary Figure 3B,D; pairwise Pearson correlation, coefficient = 0.853).

**Figure 2:**
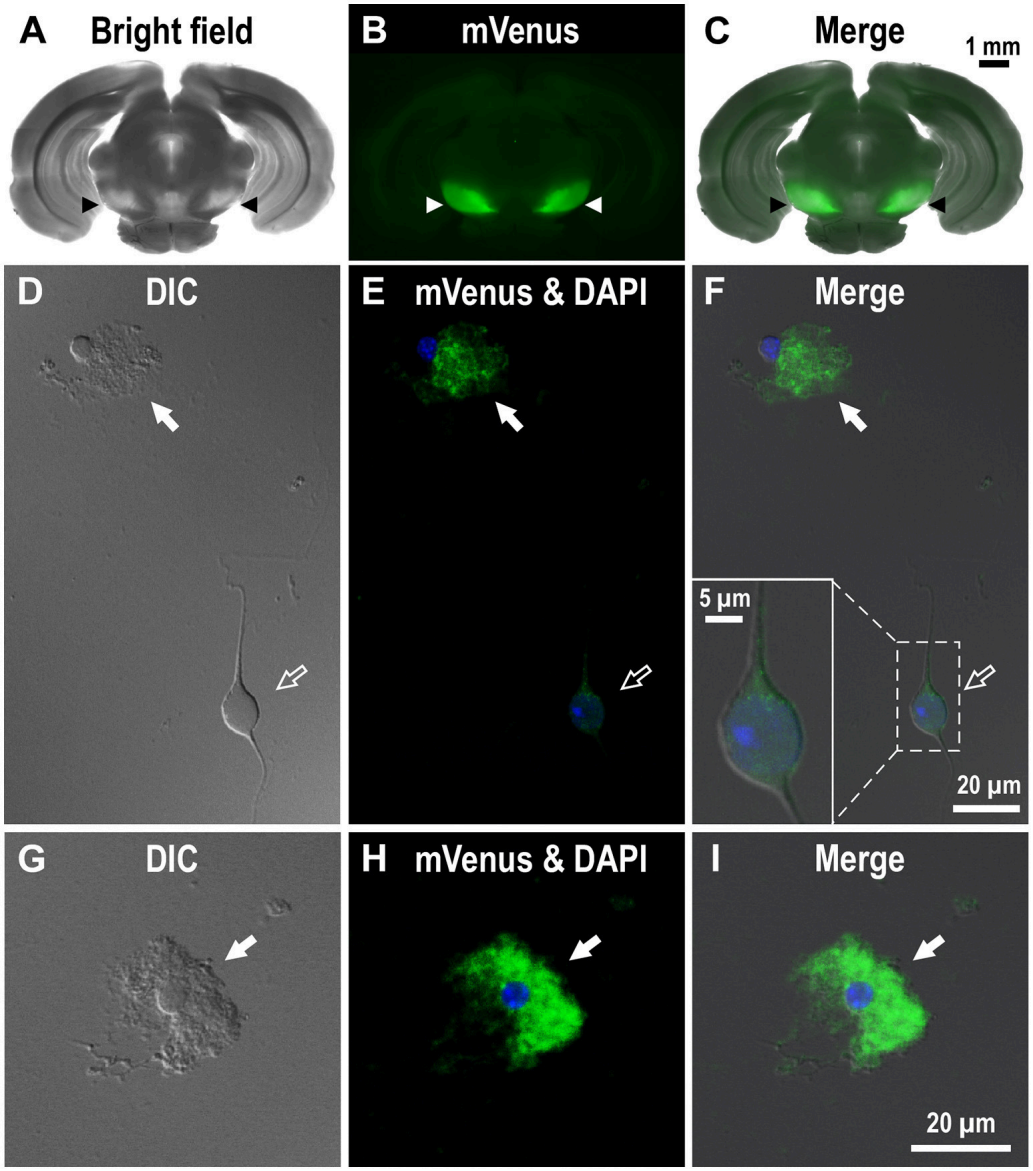
**Expression of mVenus signal in a coronal slice dissected from the Drd1-mVenus mouse brain and that in cells dissociated from the SNr of the mouse. (A–C)** Images of a 500 μm-thick coronal section, in which both SNr at the oculomotor nerve and visual cortex were included, revealing a prominent mVenus signal in the SNr (arrowheads). **(A)**, **(B)**, and **(C)** show a bright field image, mVenus fluorescence, and a merged image, respectively. Note the considerable difference in mVenus fluorescence between SNr and visual cortex. **(D–F)** Cells acutely dissociated from the SNr of adult Drd1-mVenus mouse. **(D)** Differential interference contrast image. **(E)** A glia-like cell bearing mVenus-positive fine processes (filled arrow) and a neuron exhibiting a very weak mVenus fluorescence (empty arrow). **(F)** Merged image of **(D,E)**. Synaptic bouton-like structures showing mVenus signal (see inset for a magnified view). Nuclear staining with DAPI (blue) was overlaid in **(E,F)**. **(G–I)** Similar to **(D–F)**, but a typical glia-like cell bearing bushy mVenus-positive processes in a magnified view (filled arrow).

To investigate the dense D1R immunoreactivity in SNr more closely, we examined coronal brain slices of adult Drd1-mVenus mice (Nagai et al., 2016). As shown, strong mVenus fluorescence was highly localized in the SNr (Figures 2A–C, arrowheads). To identify the cellular entity emitting mVenus fluorescence, the SNr region was punched out from these slices, and the cells were then carefully dissociated so that their fine processes were preserved as far as possible, by modifying the method reported previously (Yamada et al., 2001). As demonstrated in confocal microscopic images, glia-like cells having small cell body (mean longitudinal diameter of the cell body = 8.1 ± 1.4 μm, *n* = 167) emitted prominent mVenus fluorescence, particularly in their fine processes (Figures 2D–F, filled arrows). The fluorescence intensity for the region of interest (ROI), which was set in the proximal processes, was 353.8 ± 187.8 arbitrary units (a.u., *n* = 167) on average. In contrast, larger cells bearing dendrites (mean longitudinal diameter = 17.3 ± 5.2 μm, *n* = 191), which were presumably neurons as judged from our previous patch clamp studies (Yamada et al., 2001), exhibited only weak fluorescence (Figures 2D–F, empty arrows). When the ROI was placed in the cell body of such neurons, the mean fluorescence intensity (83.3 ± 54.0 a.u., *n* = 191) was significantly weaker than that in the small glia-like cells (*p* < 0.0001, unpaired *t*-test), although extremely small (<1 μm), synaptic bouton-like fluorescent dots were found on the dendrites and/or the cell body in 142 of the 191 cells examined (see inset in Figure 2F). Another mVenus-expressing cell shown in magnified view depicts a typical small cell bearing “bushy” mVenus-positive processes (Figures 2G–I, filled arrows). These results suggest D1R expression in the glia-like cells in the SNr.

**Figure 3:**
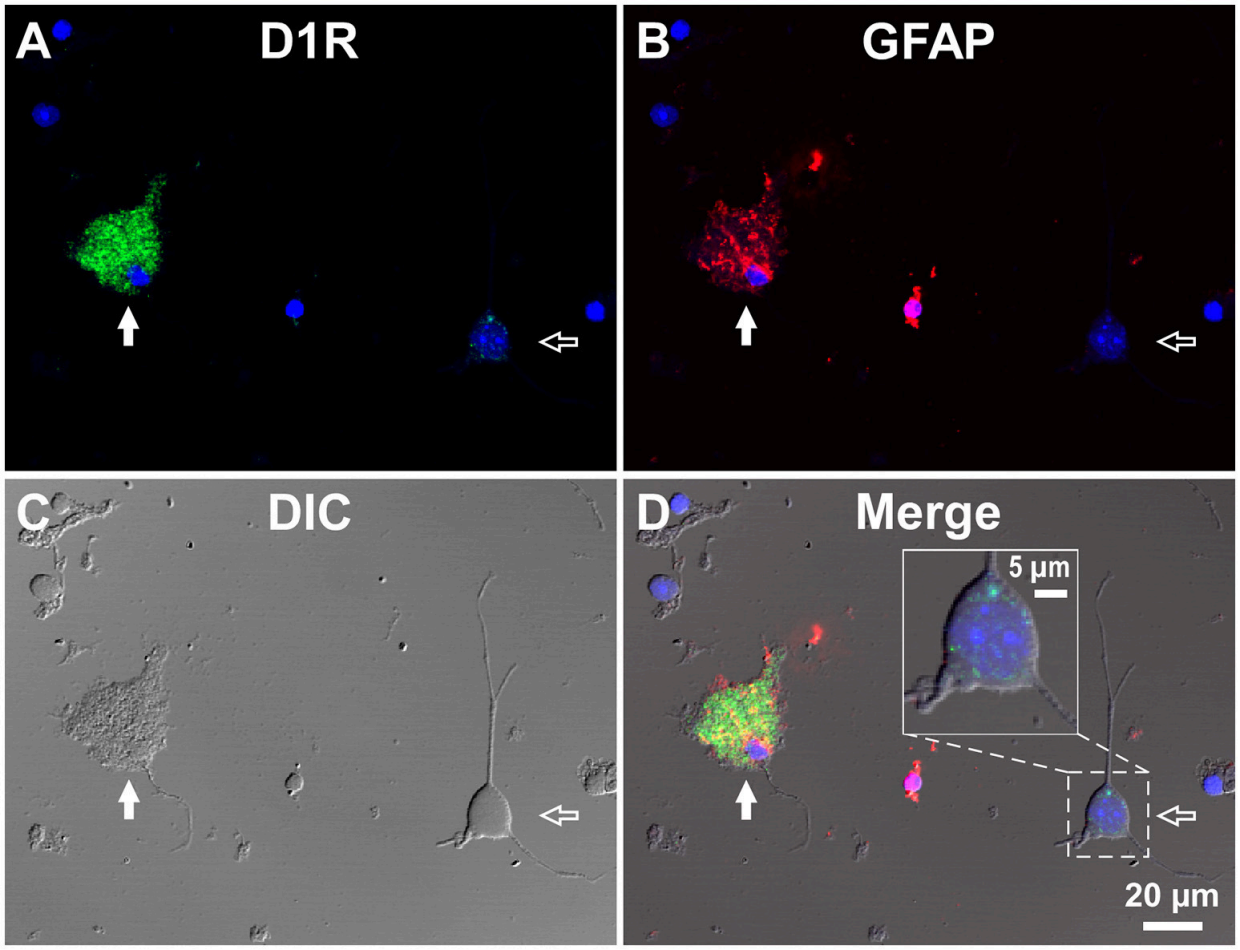
**Double immunolabeling of SNr cells dissociated from the adult wild-type mouse brain with anti-D1R and anti-GFAP antibodies. (A–D)** A typical D1R-positive/GFAP-positive SNr astrocyte (filled arrow). A typical D1R-negative (GFAP-negative) SNr neuron is shown in the same field of view (empty arrow). **(A)**, **(B)**, **(C)**, and **(D)** represent represent D1R immunofluorescence, GFAP immunofluorescence, differential interference contrast image, and a merged image of these, respectively. Nuclear staining with DAPI (blue) was overlaid. Inset in **(D)** depicts a magnified view of D1R-positive synaptic bouton-like structures on the SNr neuron. D1R-negative small cells bearing no fine process were excluded from the analysis due to too much digestion and/or mechanical damage.

To investigate whether astrocytes are involved in the D1R-expressing, glia-like cells in the SNr, we conducted double immunofluorescence imaging of SNr cells dissociated from the brains of eight adult wild-type mice with anti-D1R antibody in combination with an antibody against GFAP, a specific marker for astrocytes. The results show remarkable D1R immunoreactivity in most GFAP-positive astrocytes examined (Figures 3A–D; see also Supplementary Figure 4). The mean longitudinal diameter of the GFAP-positive cell bodies (8.0 ± 1.1 μm, *n* = 52) obtained from the SNr was similar to that of the mVenus-expressing glia-like cells in this nucleus. The mean fluorescence intensity of D1R immunoreactivity in these GFAP-positive cells was 1662.5 ± 691.9 a.u. (*n* = 52), when ROIs were positioned similarly to those in mVenus-expressing cells.

In contrast, GFAP-negative, larger cells bearing a few thick dendrites, presumably neurons (mean longitudinal diameter = 19.0 ± 4.5 μm, *n* = 40), exhibited only weak D1R immunoreactivity (Figures 3A–D, empty arrows), except for that of the bouton-like fluorescent structures on the cell body and/or dendrites in 23 of 40 cells (magnified view in Figure 3D inset). The mean fluorescence intensity for D1R in the neurons obtained from the SNr (541.7 ± 160.8 a.u. for a ROI positioned in the cell body, *n* = 40) was much weaker than that of the GFAP-positive astrocytes in the same nucleus (*p* < 0.0001, unpaired *t*-test; see also **Figure 6A**).

**Figure 4:**
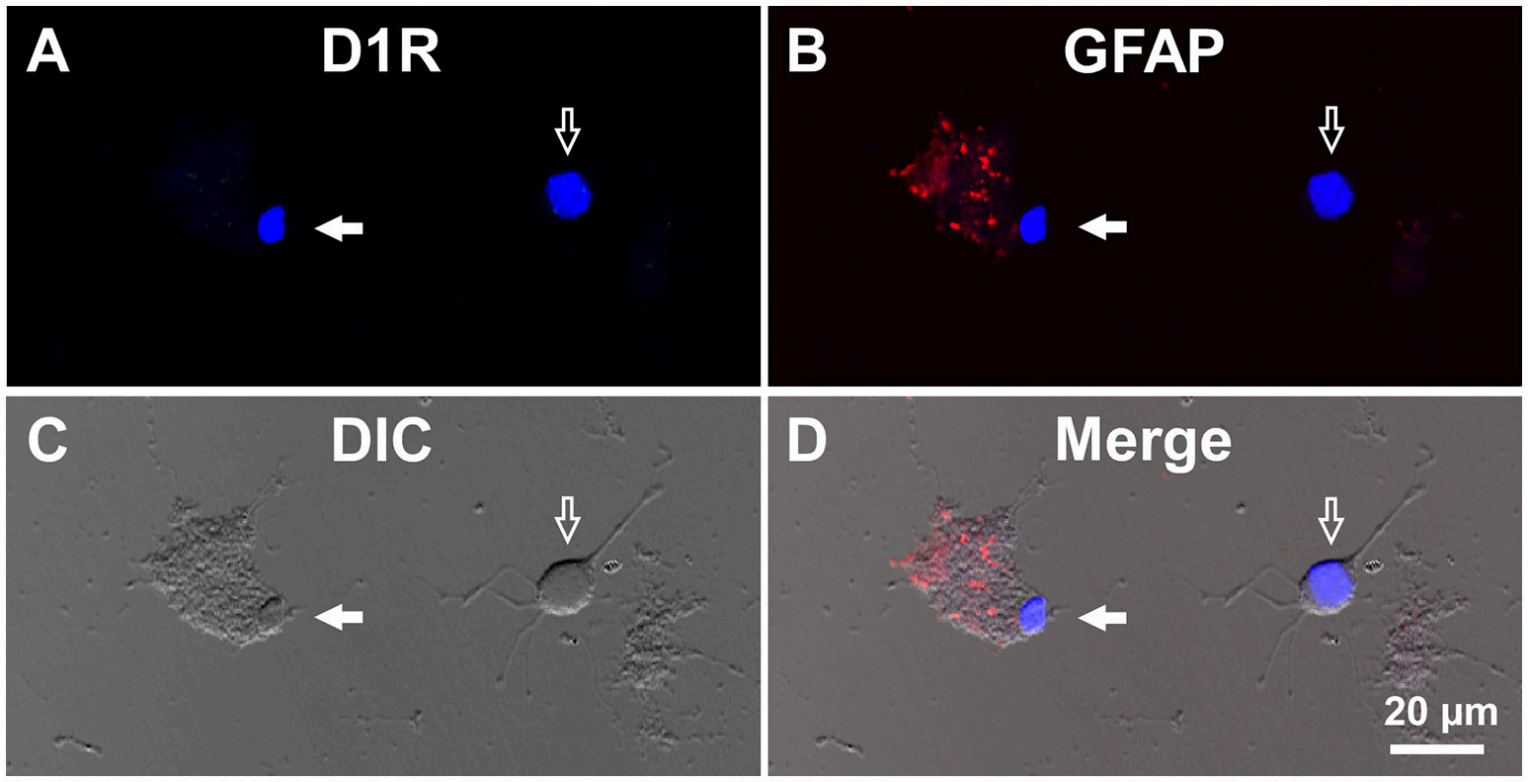
**Double immunolabeling of visual cortical cells dissociated from the adult wild-type mouse brain with anti-D1R and anti-GFAP antibodies. (A-D)** Similar to Figure 3, but for cells dissociated from the visual cortex. A typical D1R-negative/GFAP-positive visual cortical astrocyte (filled arrow). A typical D1R-negative (GFAP-negative) visual cortical neuron is shown in the same field of view (empty arrow). Nuclear staining with DAPI (blue) was overlaid in **(A,B,D)**.

For comparison, double immunocytochemistry was also conducted for visual cortical cells dissociated from the same coronal slices of the eight adult wild-type mice. As shown, GFAP-positive astrocytes obtained from the visual cortex (mean longitudinal diameter = 7.2 ± 1.0 μm, *n* = 39) showed none or only weak D1R immunoreactivity when compared in the same experimental condition as with the SNr cells (Figures 4A–D, filled arrows). The mean fluorescence intensity of D1R immunoreactivity in such GFAP-positive astrocytes obtained from the visual cortex (374.4 ± 238.8 a.u., *n* = 39) was significantly weaker than that of the GFAP-positive SNr astrocytes (*p* < 0.0001, unpaired *t*-test; see also **Figures 6A,B**, red triangles). Neurons dissociated from the visual cortex of the same slices also showed a small D1R immunoreactivity (Figures 4A–D, empty arrows; mean fluorescence intensity = 371.4 ± 171.9 a.u., *n* = 150), although the diameter of the neurons examined was relatively small on average (12.9 ± 2.4 μm, *n* = 150; see also **Figure 6B**) compared to that of SNr neurons (*p* < 0.0001, unpaired *t*-test).

**Figure 5:**
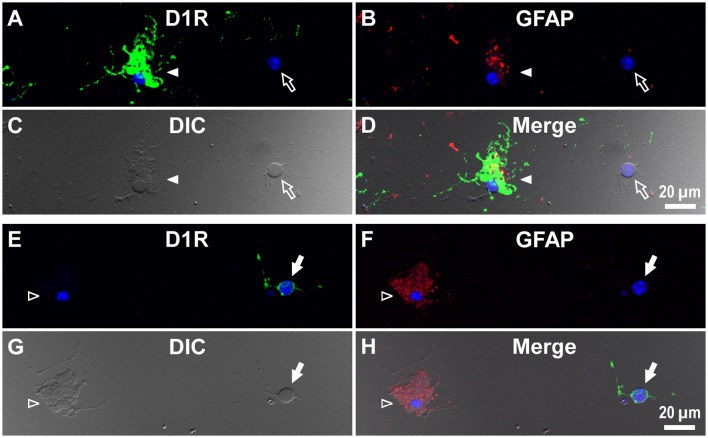
**Double immunolabeling of striatal cells dissociated from the adult wild-type mouse brain with anti-D1R and anti-GFAP antibodies. (A–D)** Similar to Figure 4, but for cells dissociated from the striatum. A typical D1R-positive/GFAP-positive striatal astrocyte (filled arrowhead) is shown. A typical D1R-negative (GFAP-negative) striatal neuron is also shown in the same field of view (empty arrow). In contrast to **(A–D)**, **(E–H)** show a typical D1R-negative/GFAP-positive striatal astrocyte (empty arrowhead). A typical D1R-positive (GFAP-negative) striatal neuron is also shown in the same field of view (filled arrow). Nuclear staining with DAPI (blue) was overlaid except for **(C,G)**.

**Figure 6:**
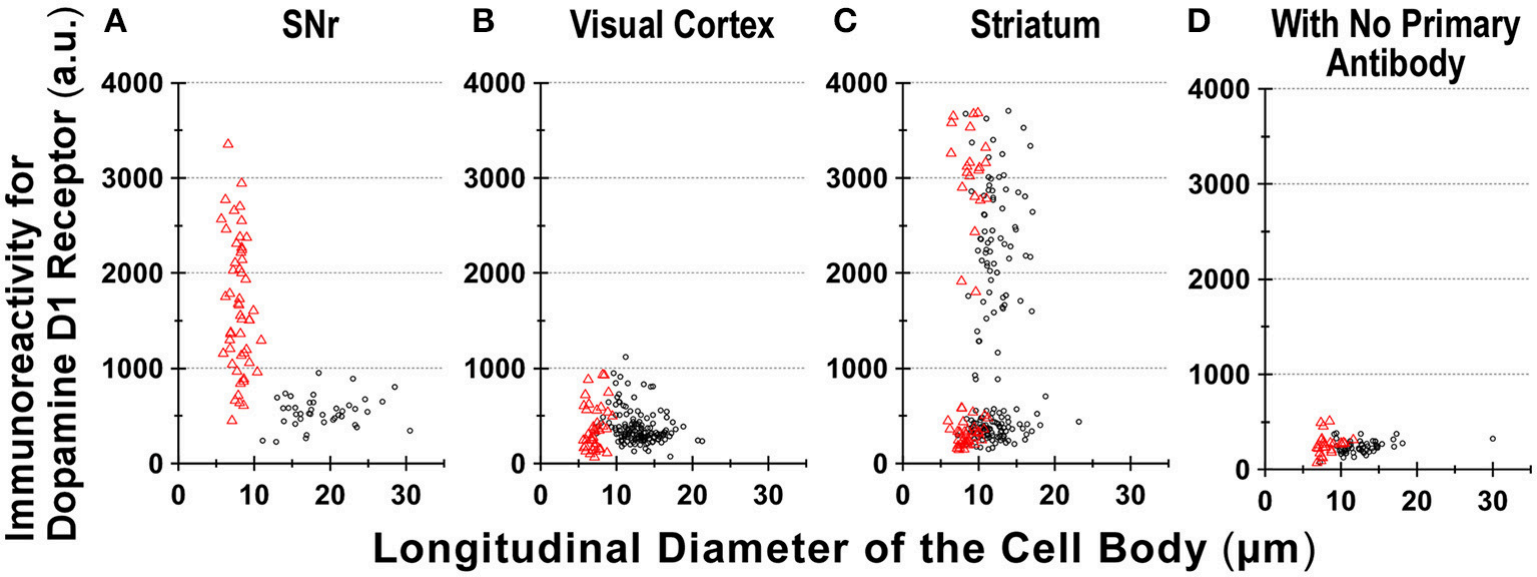
**Divergence in D1R immunoreactivity of cells acutely dissociated from SNr, visual cortex, and striatum of the adult wild-type mouse brain. (A–D)** The intensity profiles of D1R immunofluorescence, which were measured for isolated cells from SNr **(A)**, visual cortex **(B)**, and striatum **(C)**, were summarized against the longitudinal diameter of the cell body. Red triangles and black circles represent astrocytes and neurons, respectively. **(D)** Fluorescence intensity of cells acutely dissociated from SNr, visual cortex, and striatum, to which only Alexa Fluor 488 donkey anti-goat IgG and Cy3-conjugated donkey anti-rabbit IgG were applied, without using primary antibodies such as goat polyclonal anti-mouse D1R antibody and polyclonal rabbit anti-GFAP antibody.

Interestingly, when we examined cells in the striatum of a wild-type mouse in a similar manner, we noticed that GFAP-positive astrocytes tended to express either abundant D1R immunoreactivity (Figure 6C), as exemplified in Figures 5A–D (filled arrowheads), or none or only a minimum amount (Figure 6C), as shown in Figures 5E–H (empty arrowheads).

We also detected D1R-negative (Figures 5A–D, empty arrows) as well as D1R-positive (Figures 5E–H, filled arrows) neurons in the striatum in the same preparation (Figure 6C; see also Supplementary Figures 5). Similar results were obtained in duplicated experiments, implying divergent expressions of D1R in GFAP-positive astrocytes in the striatum, possibly in a manner similar to that in neurons in this nucleus in our experimental condition (Figure 6C). This tendency was supported by a cluster analysis as well (Ward's method, data not shown), although much larger sampling is required for confirming this tendency.

## Discussion

For understanding the role of dopamine in the brain, precise information on the localization of the five dopamine receptor subtypes would be of primary importance (Beaulieu and Gainetdinov, 2011). The expression of D1R has long been studied, as is also the case with that of D2R (Levey et al., 1993; Smiley et al., 1994; Yung et al., 1995; Caille et al., 1996; Kliem et al., 2010; Shao et al., 2013). It is now well established that SNr and striatum are among major brain nuclei in which D1R is most abundantly expressed (Levey et al., 1993; Yung et al., 1995; Caille et al., 1996; Kliem et al., 2010; for reviews see Fallon and Loughlin, 1995; Rommelfanger and Wichmann, 2010). SNc dopaminergic neurons transmit dopamine on the one hand through their axons to the striatum, and on the other hand through their dendrites to the SNr (Geffen et al., 1976; Korf et al., 1976; Nieoullon et al., 1977; Reubi et al., 1977; Cheramy et al., 1981). However, in the SNr, possibly due to the non-axonic nature of the dopamine transmission, the cellular entity receiving the dopamine has yet to be completely identified.

We demonstrate in the present study that GFAP-immunopositive astrocytes, which were acutely dissociated from the SNr of the adult wild-type mouse, bore fine processes showing prominent D1R immunoreactivity. Judging from the staining patterns of the wild-type mouse brains (Supplementary Figure 1B) as well as the inability of staining D1R-KO brains, the anti-D1R antibody used appeared highly reliable, leading us to propose that D1R is expressed in fine processes of GFAP-positive astrocytes in the SNr of the adult mouse brain. This concept was further supported by the results using D1R promotor-controlled mVenus expressing mice.

Region-specific astrocyte heterogeneity has been a subject of intensive studies (Oberheim et al., 2012). Our results suggest heterogeneity of D1R immunoreactivity among the GFAP-positive astrocytes obtained from three areas of the adult mouse brain (Figure 6). Although some sampling bias cannot be excluded, it would be difficult to explain such heterogeneity in D1R immunoreactivity of GFAP-positive astrocytes solely by reactive responses that might be caused by the dissociation procedure, since the quantification was made among astrocytes according to the same experimental condition (see Materials and Methods). Indeed, the coronal section of Drd1-mVenus mouse brain showed extensive heterogeneity in the D1R promotor-controlled fluorescence signal (Figure 2C). Consistently, most SNr astrocytes were more or less immunopositive for D1R (Figure 6A), whereas the cortical astrocytes tested (Figure 6B) showed only little D1R immunoreactivity.

The small diameter (12.9 ± 2.4 μm, Figure 6B) of the neurons dissociated from the visual cortex suggests difficulty in isolating large pyramidal neurons from adult mouse brain. The neurons dissociated might correspond to cells such as pyramidal neurons reported in the middle layer of the mouse visual cortex (Gilman et al., 2016). Previous studies in monkey prefrontal cortex have reported D1R immunoreactivity on pyramidal neurons (Smiley et al., 1994). Although species difference between primate and rodent cannot be excluded from responsibility for the discrepancy (Levey et al., 1993; Smiley et al., 1994), the specificity and sensitivity of the anti-D1R antibodies tested are of particular importance for interpretation of the immunoreactivity (Bergson et al., 1995; Puighermanal et al., 2016).

Further study is needed for elucidating functional roles of the divergent D1R expression in astrocytes in correlation with the well-known heterogeneity of neurons in individual brain areas (Levey et al., 1993; Fallon and Loughlin, 1995). It may be of interest that the striatal astrocytes tended to show either strong or minimum D1R immunoreactivity, considering that the principal neurons (medium spiny neurons) in the striatum are either positive or negative for D1R (Yung et al., 1995). Investigation for potential contributions of other glial cells, such as microglia, in the D1R expression in the SNr, should also be considered (Färber et al., 2005; Pannell et al., 2014).

So far, evidence for D1R expression in mature astrocytes has been largely lacking, although astrocytes expressing other dopamine receptors have been reported by several groups. Kliem et al. reported occasional expression of dopamine D5 receptor (D5R) in some glial processes in monkey SNr and internal globus pallidus by immunoelectronmicroscopy (Kliem et al., 2010). In prefrontal cortex, D2R expression in astrocytes has been reported as well (Khan et al., 2001; Negyessy and Goldman-Rakic, 2005; Duffy et al., 2011). Using mice lacking D2R preferentially in GFAP-expressing cells, Shao et al. reported D2R expression in astrocytes and its role in suppressing 1-methyl-4-phenyl-1,2,3,6-tetrahydropyridine (MPTP)-induced neurotoxicity (Shao et al., 2013). Bosson et al. reported recently that disruption of dopaminergic transmission can remodel astrocytic calcium activity within the SNr (Bosson et al., 2015).

It is well established that the major function of SNr is to regulate motor activity by inhibiting remote nuclei, superior colliculus, thalamus, and pedunculopontine nucleus of the brain stem according to inputs from the striatum (Hikosaka et al., 2000, 2014; Takakusaki et al., 2004). In addition, some groups including ours have suggested that SNr contributes to sensing hypoxic/hypoglycemic conditions (Yamada et al., 2001; Yuan et al., 2004; Yamada and Inagaki, 2005; Velisek et al., 2008; Lutas et al., 2014). Since fine astrocyte processes contain very small mitochondria (Derouiche et al., 2015), dopamine might well alter metabolic activity of astrocytes at the fine processes (Requardt et al., 2010).

Brain circuits have been largely discussed based on neuron to neuron connections that convey signals mediated by such as axo-dendritic and axo-axonic transmissions. Recently, we reported that astrocytes obtained from the immature mouse cortex can release glycine in response to dopamine through reverse operation of glycine transporter 1, in which D1-like receptors might be involved (Shibasaki et al., 2017). Thus, it may be of interest to explore whether astrocytes are associated with the dopamine function in the SNr. Reubi and Sandri, who examined 38 blocks and 24 freeze-etching replica at the electronmicroscopic level, reported that nigral dendrites are consistently separated by one or two thin glial sheaths (Reubi and Sandri, 1979). Since strong D1R immunoreactivity was found in fine processes of the SNr astrocyte, the astrocyte might well be a major candidate to receive dopamine released dendritically. Further study is required to confirm the present data at the electronmicroscopic level and to elucidate the functional role of divergent dopamine receptor immunoreactivity in astrocytes.

## Author contributions

KN carried out and analyzed all single cell experiments and wrote the manuscript; SS invented the dissociation procedure and analyzed the data; MS and YY performed immunohistochemistry; YY participated in the discussion; MK produced animals including D1R-KO and Drd1-mVenus; KK developed Drd1-mVenus and participated in the discussion; KY designed, organized and analyzed the experiments, and wrote the manuscript.

## Funding

This study was supported by Grants-in-Aid for Scientific Research (KAKENHI) 17590182 (KY), 23650203 (KY), 26860143 (KN); The Cooperative Study Program of National Institute for Physiological Sciences No.12, 22, 34, and 226 (KY); Hirosaki University Institutional Research Grant (KY).

### Conflict of interest statement

The authors declare that the research was conducted in the absence of any commercial or financial relationships that could be construed as a potential conflict of interest.
