# Supplementary material for: Dopamine D1 Receptor Immunoreactivity on Fine Processes of GFAP-Positive Astrocytes in the Substantia Nigra Pars Reticulata of Adult Mouse
Source: Front Neuroanat. 2017 Feb 1;11:3. doi: 10.3389/fnana.2017.00003 (PMC5285371; doi:10.3389/fnana.2017.00003)
Supplement: Supplementary file 1 [file Image1.PDF]

SUPPLEMENTARY FIGURE 1

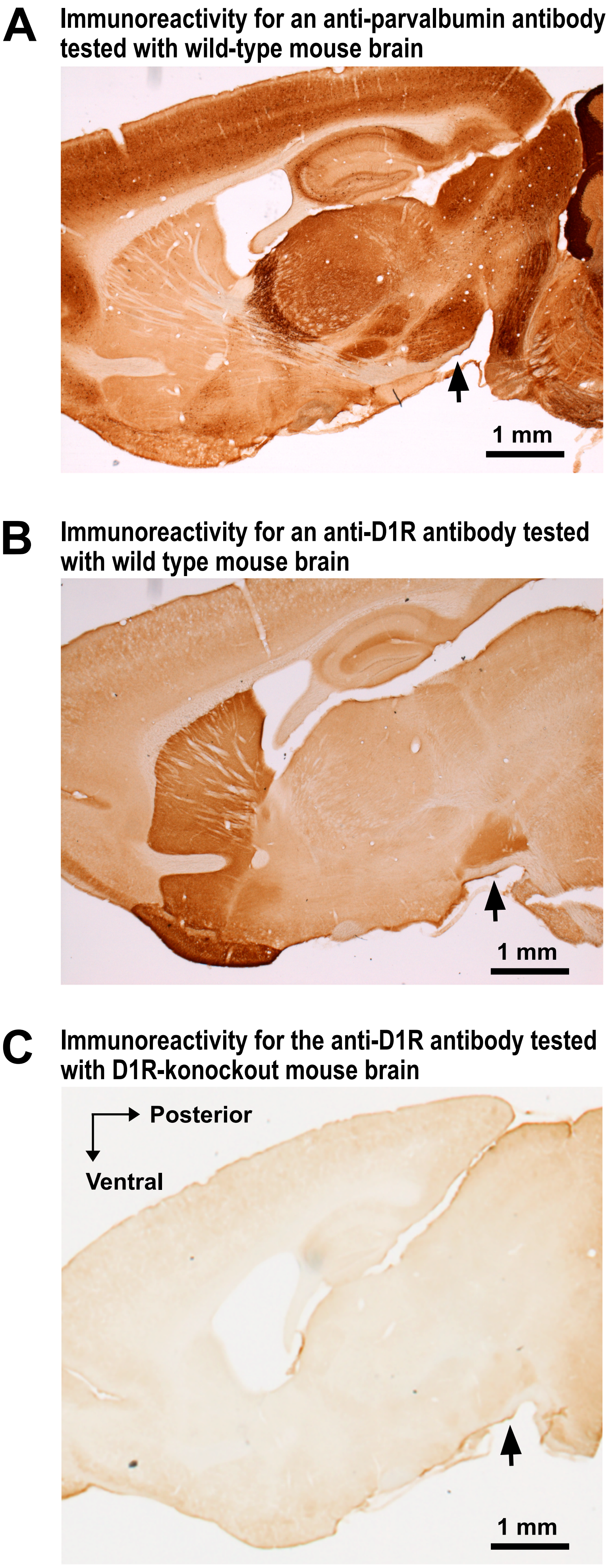

SUPPLEMENTARY FIGURE 1 | Immunoreactivity of anti-D1R antibodies examined for wild-type and D1R-KO mouse brains in sagittal sections.
(A) Immunolabeling of the wild-type mouse brain with anti-parvalbumin antibody performed similarly to in **Figure 1B**, but in a sagittal section including the SNr (arrow). **(B)** Immunoreactivity of the anti-D1R antibody for the wild-type mouse brain in a sagittal section including the SNr (arrow). **(C)** Similar to **(B)**, but for D1R-KO mouse brain, demonstrating no appreciable immunoreactivity in the SNr (arrow). Orientations are common to A-C.
