# Supplementary material for: Dopamine D1 Receptor Immunoreactivity on Fine Processes of GFAP-Positive Astrocytes in the Substantia Nigra Pars Reticulata of Adult Mouse
Source: Front Neuroanat. 2017 Feb 1;11:3. doi: 10.3389/fnana.2017.00003 (PMC5285371; doi:10.3389/fnana.2017.00003)
Supplement: Supplementary file 2 [file Image2.PDF]

Nagatomo et al.

SUPPLEMENTARY FIGURE 2

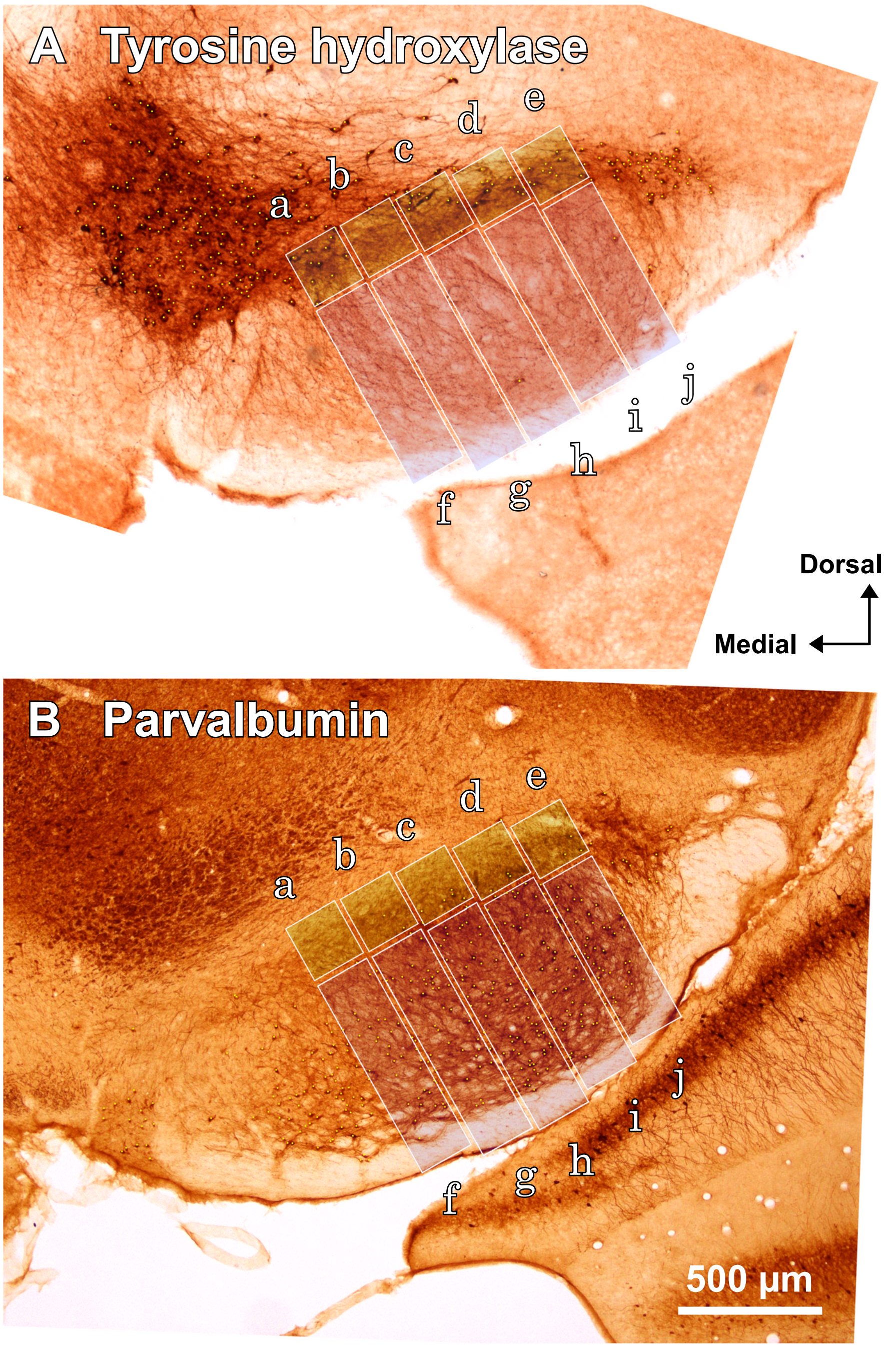

SUPPLEMENTARY FIGURE 2 | Comparison of the number of tyrosine hydroxylase (TH)-immunopositive dopaminergic neurons and parvalbumin (Parv)-immunopositive GABAergic neurons in substantia nigra pars compacta (SNc) and pars reticulata (SNr).
Coronal brain sections including substantia nigra immunolabeled with anti-TH antibody (A, the same as Figure 1D) and anti-Parv antibody (B, the same as Figure 1B). The dorsolateral part of the substantia nigra was subdivided into five regions of interest (ROIs) in SNc (faint green squares) and in SNr (faint blue rectangles), respectively. SNc and SNr were determined based on Paxinos, G. and Franklin, K.B.J. "The mouse brain in stereotaxic coordinates." 2nd edition, 2001 in serial sections. A, TH-immunopositive cell bodies were marked by yellow dots throughout the section. The number of TH-immunopositive cell bodies included in the ROI was counted for each ROI, and statistically compared between SNc (a to e) and SNr (f to j) with student unpaired *t*-test after conducting particle analyses with Fiji. Data represented are mean ± S.D. (the number of cell bodies/10000 μm^2) The number of TH-immunopositive cells was significantly larger in SNc (mean =2.23 ± 0.70, n = 5) than that in SNr (mean =0.04 ± 0.07, n = 5; $p < 0.003$) . B, Similar to (A), but for Parv-immunopositive cell bodies. The number of Parv-immunopositive GABAergic neurons was significantly larger in SNr (mean = 2.97 ± 0.71, n = 5) than in SNc (mean = 0.71 ± 0.53, n = 5, $p < 0.001$). Similar results were obtained in two separate experiments, indicating that Parv-immunopositive GABAergic neurons are more abundant in SNr than those in SNc. The orientation and the scale are common to A and B.
