# Supplementary material for: Dopamine D1 Receptor Immunoreactivity on Fine Processes of GFAP-Positive Astrocytes in the Substantia Nigra Pars Reticulata of Adult Mouse
Source: Front Neuroanat. 2017 Feb 1;11:3. doi: 10.3389/fnana.2017.00003 (PMC5285371; doi:10.3389/fnana.2017.00003)
Supplement: Supplementary file 3 [file Image3.PDF]

SUPPLEMENTARY FIGURE 3

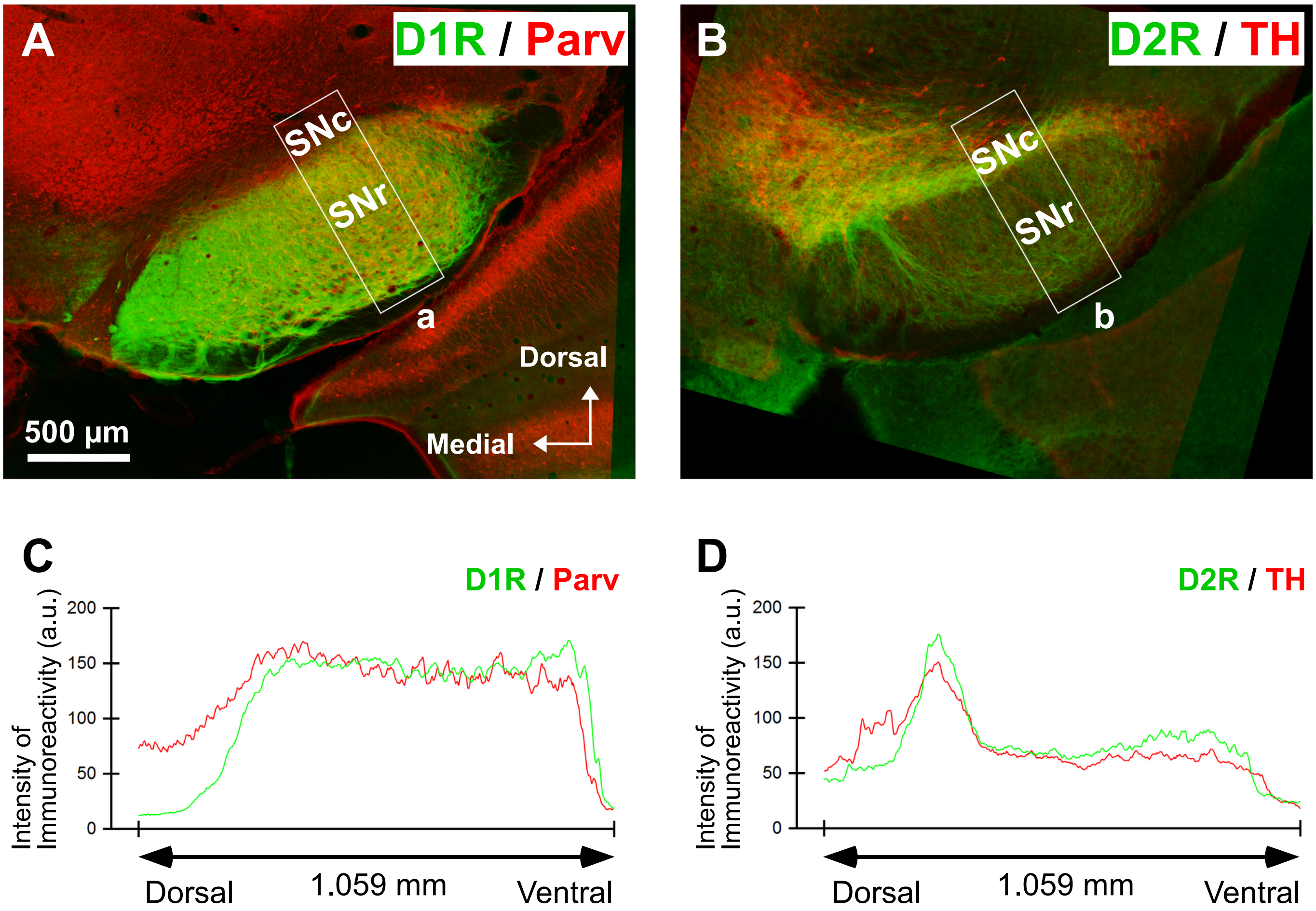

SUPPLEMENTARY FIGURE 3 | Comparison of immunoreactivity for D1R / parvalbumin (A,C) and D2R / TH (B,D) in substantia nigra in coronal section
(A) D1R-Immunolabeled section (Figure 1A) was overlaid with parvalbumin (Parv)-immunolabeled adjacent section (Figure 1B). Original DAB images were color-inverted after optimization of contrast and brightness, grayscaled, and pseudo-colored to green (D1R) and red (Parv), then overlaid. (B) Similar to (A), but D2R-immunolabeled section (Figure 1C) was overlaid with tyrosine hydroxylase (TH)-immunolabeled adjacent section (Figure 1D) and pseudo-colored to green (D2R) and red (TH). (C) Intensity of pseudo-colored immunoreactivity for D1R (green line) and that for Parv (red line) were plotted for the boxed region (a) from the SNc to the SNr direction using IGOR PRO ver.7 (Line profile width=150). (D) Similar to (C) but processed for D2R and TH. Pairwise Pearson correlation was calculated using JMP 11.2. after normalizing the individual intensity. The correlation coefficient between D1R and Parv was 0.808, and that beween D2R and TH was 0.853. The orientation and the scale are common to A and B.
