# Supplementary material for: Dopamine D1 Receptor Immunoreactivity on Fine Processes of GFAP-Positive Astrocytes in the Substantia Nigra Pars Reticulata of Adult Mouse
Source: Front Neuroanat. 2017 Feb 1;11:3. doi: 10.3389/fnana.2017.00003 (PMC5285371; doi:10.3389/fnana.2017.00003)
Supplement: Supplementary file 4 [file Image4.PDF]

SUPPLEMENTARY FIGURE 4

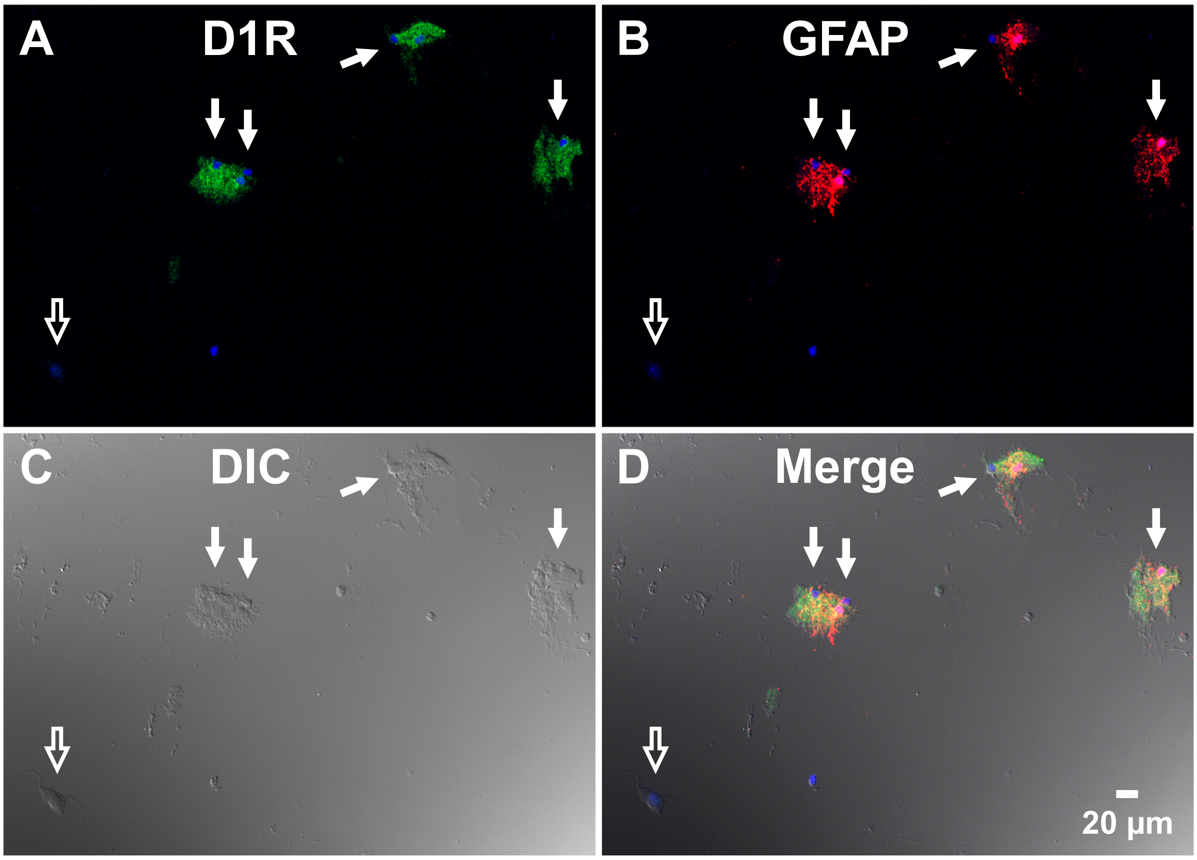

SUPPLEMENTARY FIGURE 4 | Typical D1R-positive/GFAP-positive astrocytes dissociated from the SNr of the adult wild-type mouse brain in a low magnification view. (A-D) GFAP-positive astrocytes showing significant immunoreactivity for anti-D1R antibody (filled arrows). A D1R-negative (GFAP-negative) SNr neuron was shown as well in the same field of view (empty arrow). (A), (B), (C), and (D) represent D1R immunofluorescence, GFAP immunofluorescence, differential interference contrast image, and a merged image of these, respectively.
