# Supplementary material for: Dopamine D1 Receptor Immunoreactivity on Fine Processes of GFAP-Positive Astrocytes in the Substantia Nigra Pars Reticulata of Adult Mouse
Source: Front Neuroanat. 2017 Feb 1;11:3. doi: 10.3389/fnana.2017.00003 (PMC5285371; doi:10.3389/fnana.2017.00003)
Supplement: Supplementary file 5 [file Image5.PDF]

SUPPLEMENTARY FIGURE 5

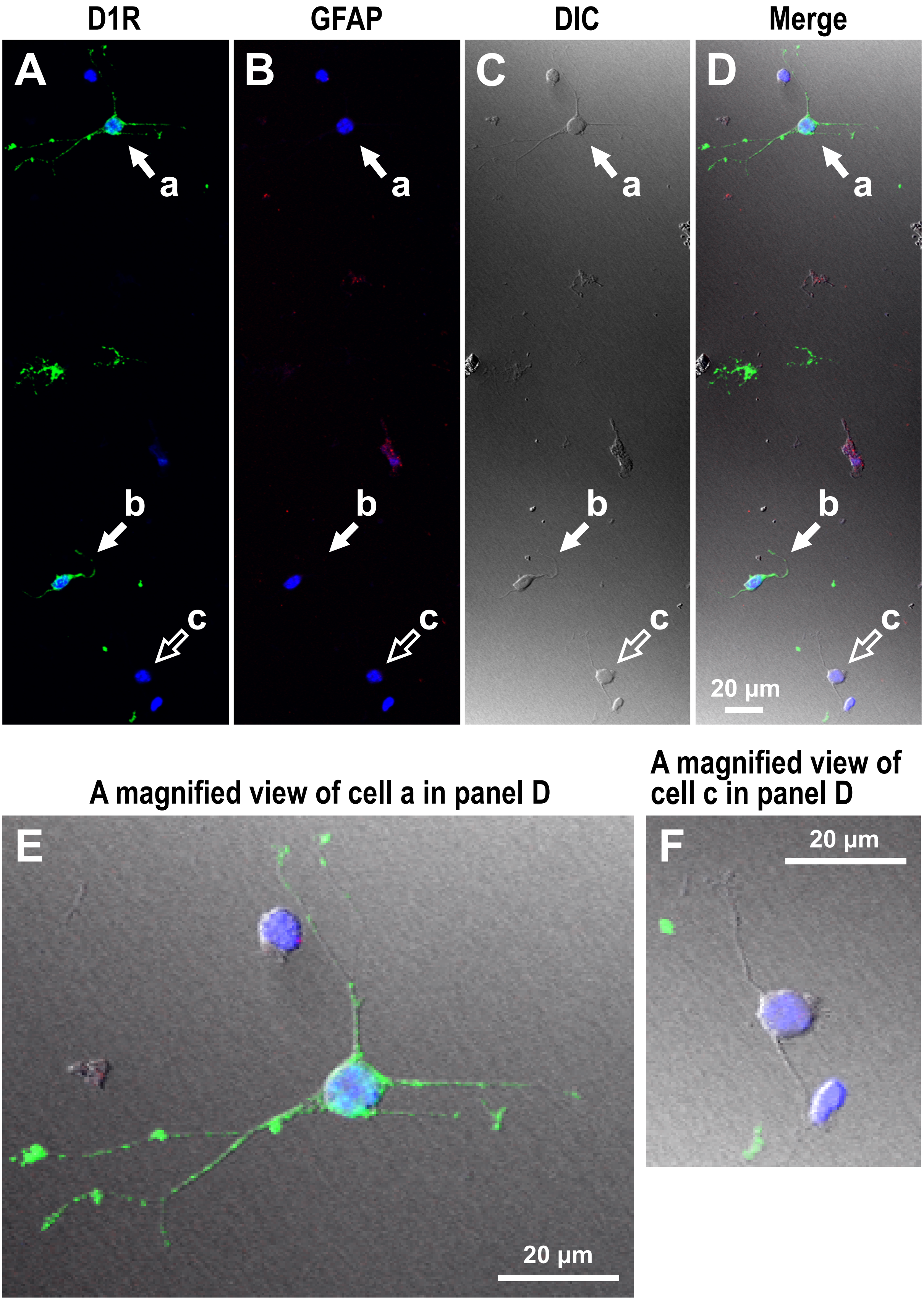

SUPPLEMENTARY FIGURE 5 | Immunoreactivity of the anti-D1R antibody examined for typical striatal neurons of the adult wild-type mouse brain.
(A-D) Similar to Figure 5, but another typical example of D1R-positive (GFAP-negative) (filled arrows) and D1R-negative (GFAP-negative) (empty arrow) striatal neurons. (E) and (F) represent magnified views of cell a and c in panel (D), respectively.
